# Serum C-reactive protein in asthma and its ability in predicting asthma control, a case-control study

**Published:** 2016

**Authors:** Mahmoud Monadi, Alireza Firouzjahi, Amin Hosseini, Yahya Javadian, Majid Sharbatdaran, Behzad Heidari

**Affiliations:** 1 Department of Internal Medicine, Rouhani Hospital, Babol University of Medical Sciences, Babol, Iran.; 2 Department of Laboratory Medicine, Rouhani Hospital, Babol University of Medical Sciences, Babol, Iran.; 3 Mobility Impairment Research Center, Babol University of Medical Sciences, Babol, Iran.

**Keywords:** Asthma, Asthma control, High sensitive C-reactive protein, Prediction

## Abstract

**Background:**

Increased serum high sensitive C-reactive protein (hs-CRP) in asthma and its association with disease severity has been investigated in many studies. This study aimed to determine serum hs-CRP status in asthma versus healthy controls and to examine its ability in predicting asthma control.

**Methods:**

Serum CRP was measured by ELISA method using a high sensitive CRP kit. Severity of asthma was determined using Asthma Control Test. Spearman and chi square tests were used for association and correlation respectively. The predictive ability was determined by receiver operating characteristics (ROC) analysis. Accuracy was determined by determination of area under the ROC curve (AUC).

**Results:**

A total of 120 patients and 115 controls were studied. Median serum hs-CRP in asthma was higher than control (P=0.001. In well controlled asthma the hs-CRP decreased significantly compared with poorly controlled (P=0.024) but still was higher than control (P=0.017). Serum hs-CRP at cutoff level of 1.45 mg/L differentiated the patients and controls with accuracy of 63.5 % (AUC= 0.635±0.037, P=0.001). Serum hs-CRP ≤ 2.15 mg/L predicted well controlled asthma with accuracy of 62.5% (AUC= 0.625±0.056, p=0.025). After adjusting for age, sex, weight and smoking, there was an independent association between serum hs-CRP >1.45 mg/L and asthma by adjusted OR=2.49, p=0.018).

**Conclusion:**

These findings indicate that serum hs-CRP in asthma is higher than healthy control and increases with severity of asthma and decreases with. Thus, serum hs-CRP measurement can be helpful in predicting asthma control and treatment response.

Systemic inflammation and reversible airflow narrowing is the dominant physiological feature of asthma (1). Existence of inflammation is not limited to severe asthma, but even mild and moderate asthma are also associated with inflammation of the airway wall with abnormal accumulation of inflammatory cells and airway hyper-responsiveness (2). Structural changes due to remodeling process result in irreversible airway narrowing and incompletely reversible airways' dilatation, bronchial hyper-responsiveness, airway edema, and hyper secretion. These changes are representative of severe inflammation and predispose patients to disease exacerbation and even death (2, 3). Existence of inflammatory process in asthma and its relationship with disease severity as well as deterioration of pulmonary function has been shown in several studies (4-8). Inflammation plays an important role in the pathogenesis and progression of asthma (2, 8), and thus, treatment of asthma is targeted to suppression of inflammatory process to achieve clinical response.

Efficiency of treatment in asthma is based on clinical examination and pulmonary function test. Furthermore, suppression of inflammation with appropriate treatment is also associated with reduction of serum hs-CRP level (5, 9). These observations provide a rational for serum hs-CRP to be considered as a means for detection of systemic inflammation, response to treatment as well as for estimating asthma status. Response to treatment of asthma is evaluated by Asthma Control Test (ACT) (10) but variations in clinical features which constitute the components of the ACT criteria do not usually correlate with extent of pulmonary airway structural changes (2,3).This issue warrants an insured measurable mean for the evaluation of both severity of clinical features and inflammatory process. The ability of several bio markers has been tested for diagnosis, evaluation of treatment, or prognostic purposes in asthma as well as in chronic obstructive pulmonary disease (COPD) (11-13). 

In patients with asthma the potential of serum CRP, SAA and fibrinogen in recognizing local or systemic inflammation has been shown (14) specially serum CRP which is a known parameter of inflammatory process, is sensitive to changes and readily accessible with low cost. In particular, the high-sensitive method of serum CRP measurement (hs-CRP) can detect minimal variations of serum CRP levels even in the range of normal limits, and thus can be considered as a sensitive marker for the identification of low grade systemic inflammation. The serum CRP has been used for evaluation of treatment in chronic pulmonary airway disease and other inflammatory conditions (4, 15-20. The reliability of hs -CRP as an inflammatory marker of asthma has been confirmed in correlation with sputum eosinophils (4, 7, and 21). Nonetheless, the ability of serum hs-CRP in predicting asthma control has not been investigated yet. For these reasons, the present case-control study was designed to determine the serum hs-CRP status in asthma versus healthy control and to examine the relationship between hs-CRP and asthma control determined by ACT criteria.

## Methods

The study patients were derived among patients with asthma presented for follow -up examination in outpatient pulmonary clinic of Rouhani Hospital, a university-affiliated teaching hospital in Babol, North of Iran. The patients were on maintenance treatment including inhaled corticosteroids and bronchodilators according to the guideline treatment of asthma (22). All eligible patients were included except those with COPD, bronchiectasis, pulmonary infection, connective tissue diseases, vasculitis, coexistent acute or chronic localized or systemic infection or inflammatory conditions at respiratory or musculoskeletal, gastrointestinal, urinary tract and gastrointestinal systems as well as patients with malignancies and history of inflammatory disease. The subjects of the control group were selected among the healthy personals of the same hospital that had not asthma. Similar exclusion criteria were also applied to the control group. All participants gave informed consent and the proposal of this study was approved by the Ethics Committee of Babol University of Medical Sciences, Babol, Iran. Sample size was calculated based on standard deviation of 2.9 for serum hs- CRP in asthma patients (17). At least 100 participants for each group were required to detect of 1.2 mg/L difference in serum hs-CRP concentration between patients and healthy controls with power of 80% and confidence level of 95. Twenty percent greater participants were included to compensate dropout rate. Data regarding age, sex, weight, smoking, duration of disease and treatment, frequency of symptoms, medications and previous illness and pulmonary function test were provided at the time of inclusion. Diagnosis of asthma was confirmed based on clinical features and pulmonary function test. All patients received standard treatment (22) to achieve control. The level of control of asthma was determined using ACT. The validated Persian version of the ACT questionnaire (10) was administered to all patients during the previous 4 weeks. The patients were classified as, well controlled or poorly controlled according to ACT score of ≥ 20 or < 20 respectively (23). Serum CRP was measured by ELISA method according to the manufacturer’s instruction using a high sensitive CRP kit (LBL kit, Germany) which serum levels less than 1 mg/L were considered normal. 

**Statistical Analysis: **In statistical analysis the serum hs-CRP was compared between patients and controls as well as in well controlled versus poorly controlled asthma. Receiver operating characteristics (ROC) analysis was constructed to test the ability of hs-CRP in discriminating asthma from control and differentiation of well controlled from poorly controlled asthma. The optimal cutoff levels that differentiated patients from controls or well controlled asthma from poorly controlled asthma at highest sensitivity and specificity was determined using Youdens’ index Distribution of all variables were examined by measures of skewness and kurtosis. Parametric and non-parametric Mann-Whitney U tests were used for comparison of variables with and without normal distribution respectively. The chi square test with calculation of odds ratio (OR) and 95% confidence interval (95% CI) was used for association and Spearman test was used for determination of correlation.

## Results

A total of 120 patients (73% females) with asthma and 115 healthy controls (72% females) were studied (P=0.88). The mean (±SD) age of patients and controls was 48±10 and 42.5±10 years old, respectively (p=0.001). Characteristics of the patients and controls were presented in table 1. 

**Table1:** Characteristics of patients with asthma and controls

**Variables** **Mean±SD (median)**	**Patients** **n=120**	**Controls** **n=115**	**P values**
Age, year	48.1±12	42.4±9.6	0.001
Weight, kg	73.4±12.4	79.3±9	0.001
Duration of treatment ,year	7.35±2.2	-	-
Serum hs-CRP α mg/L,	2.42±2.37(1.85)	1.29±1.03 (1)	0.001
Sex, females no (%)	88 (73.3)	75(70.1)	0.34
Smoker, no (%)	13(12)	7 (17.5)	0.56

α high sensitive C-reactive protein

Serum hs-CRP in patients had a skewed distribution from 0.1 mg/L to 16 mg/L with mean value of 2.42±2.37 mg/L and median value of 1.85 mg/L. The serum hs-CRP in the control group had also a skewed distribution from 0.05 mg/L to 5.2 mg/L with a mean value of 1.29±1.03 and median value of 1 mg/L. Median serum hs-CRP in patients was significantly higher than controls (P=0.001). In poorly controlled asthma, median serum hs-CRP was significantly higher than the well controlled asthma (2.55 versus 1.6 mg/, P=0.024). Median serum hs-CRP in both groups of patients, particularly the poorly controlled asthma was significantly higher than controls (table 2). The poorly controlled group had significantly higher age and longer disease duration (table 2). The lung volumes in the poorly controlled group were significantly lower than the well controlled group (table 2). 

**Table 2:** Comparison of demographic and laboratory characteristics of asthma patients under treatment with standard therapy classified to well controlled and poor controlled asthma according to Asthma Control Test

**Variables**	**Well** **controlled asthma** **n=80**	**Poor** **controlled asthma** **n=40**	**Pvalues**
Age, years mean	47.8±13	50±9.7	0.001
Sex, female no (%)	59 (73.8)	29 (72.5)	0.92
Smokers, no (%)	12 (16.5)	1 (2.8)	0.058
Disease duration, mean, years	9.3±8(7)	12±.2 (12)	0.014
Treatment duration, mean, years	7.2±5.5 (6)	11.1±7 (12)	0.004
FEV1 %,mean	77.3±22.8	63.6±19.3	0.001
FEF25-75, mean	59.2±30	38.4±24.4	0.001
FVC, mean	85.7±19.4	76.1±19.7	0.001
*Serum high sensitive C-reactive protein mg/L, mean±SD (median)	1.98±1.7 (2.55 )	3.3±3.1( 1.6 )	0.001
Weight, kg, mean±SD	73.6±12.6	73±12.2	0.002

FEV1 %=Percent predicted forced expiratory volume in 1 second,

FEF 25-75=Forced exploratory flow rate

FVC=Forced vital capacity

**Table 3:** Correlation between increased serum high sensitive C-reactive protein (hs-CRP) levels and poorly asthma control in patients with asthma under standard treatment

**hs-CRP levels(mg/L)**	* **Correlation coefficient**	**P values**
>1.45 vs ≤ 1.45	0.095	0.30
>3 vs ≤ 3	0.199	0.03
>4 vs ≤ 4	0.274	0.002
>5 vs ≤ 5	0.295	0.001

* Spearman correlation test

## Discussion

The results of this study indicated significantly higher serum hs-CRP concentration in patients with asthma than healthy controls and a positive correlation between increasing concentration of the serum hs-CRP and severity of asthma. Inhaled corticosteroid therapy was associated with reduced serum hs-CRP and the level of serum hs-CRP exhibited a predictive ability in differentiation of asthma from healthy control as well as poorly controlled asthma from well controlled asthma with moderate accuracy. Identification of high serum hs-CRP in asthma as observed in the present study is in agreement with the findings of many earlier studies (4, 6, 15, 24). In a study by Ramirez et al., serum hs-CRP in asthma patients ranged from less than 0.5 to 14.1 mg/L, (2.1±2.9 mg/L) versus less than 0.5 mg/L in healthy individuals. In the same study, there was no significant relationship between serum hs-CRP and FEV1 (17). 

In another study by Shimoda et al., the log serum hs-CRP of 2.3 distinguished asthmatic patients from healthy controls at sensitivity of 69% and specificity of 70% (15) In contrast, Sigari et al. found no correlation between degree of systemic inflammation estimated by hs-CRP and ACT scores or FEV1 even in controlled asthma (10). Similarly, in a study of patients with relatively severe asthma, serum high-sensitively CRP did not correlate with wheeze, the National Asthma Education and Prevention Program (NAEPP) control score, FEV1, or fractional exhaled citric oxide (FeNO) (17). The results of a 9-year follow- up prospective longitudinal study of the general population did not show a relationship between systemic inflammation and decline in lung function (25) Several factors like study design, criteria applied for classification of severity of asthma or asthma control, characteristics of the study patients at the time of inclusion, measures used for estimation of inflammatory state may differently affect the results of various studies and so explain the conflicting results. Negative influence of inflammation on asthma as observed in this study, has been also shown in a number of longitudinal studies. A population based study demonstrated a significant inverse association between FEV1 and forced vital capacity with CRP in both men and women independent of smoking, asthma and body mass index (26). In another longitudinal study, a 1-standard-deviation increase in log-CRP over 13 years was associated with a -64.0 ml decline in FEV1 and FVC over the same period and persisted among lifetime never-smokers (27). Many investigators have considered serum hs-CRP as an indirect tool for evaluation, monitoring of airway inflammation, and estimation of disease severity, and response to steroid treatment in asthmatic children (4-7). The results of this study also suggest ability of hs-CRP to predict asthma control. In this study, in spite of a significant reduction in serum hs- CRP with inhaled corticosteroids, the serum hs-CRP in controlled asthma remained significantly higher than controls. These findings in agreement with other studies indicate persistence of systemic inflammation even in well controlled asthma (4, 7, 9, 21, 25, 28). 

This issue is important because elevated serum CRP not only indicates lung inflammation but also an important predictor of future development of cardiovascular events. The clinical significance of serum hs-CRP measurement in asthma is not limited to its potential in estimating disease severity or evaluating the efficacy of treatment but also provides additional data in predicting future burden regarding cardiovascular morbidity and mortality. Although the association of high serum CRP and subsequent cardiovascular events in COPD has been shown, this association in asthma has not been documented and requires further studies (13). Nonetheless, reduction of serum hs-CRP with appropriate treatment including inhaled corticosteroids is anticipated to decrease morbidities (13). This context requires further studies. 

This study has limitations regarding study design which is cross-sectional and indicates an association between high serum hs-CRP level and asthma. However, aggregated or altered CRP is damaging to lung tissues and thus serum CRP may result in further lung damages (29). On the other hand, increasing serum CRP is a consequence of inflammatory process in asthma. Although other causes such as colonization of lung by bacteria, smoking, obesity, air pollution due to increased production of interlukine-6 may also stimulate CRP production in patients with stable chronic airway diseases like COPD and asthma. Furthermore, damaged lung tissue itself may be a source of sustained signal for synthesis of CRP suggesting a possible contributive role for CRP in perpetuation of asthma (29).

Another limitation of this study is lack of steroid-naive patients. All study patients were recruited among the patients taking standard treatment containing inhaled corticosteroids. Treatment of patients with corticosteroids reduces the level of serum hs-CRP (4, 16). Consequently, the magnitude of difference in hs-CRP between the patients and controls is anticipated to be underestimated and the real value is expected to be higher than that observed in this study. In spite of strict control on exclusion criteria, nevertheless, the presence of localized inflammation or infection in a number of patients with elevated serum CRP could not be ignored. However, similar conditions may be also applicable to the control group and so the results would not be affected. 

Serum CRP measurement by high sensitive method, adequate sample size, homogeneous sources of patients and control selection and similarity of treatment, classification and serum hs-CRP measurement should be considered as strength of this study. Strength of this case- control study is the source of population from which the cases are were derived. All cases were drawn among asthmatic patients that attended to a single hospital. As a results the study patients are expected to be representative of all the cases in the population. The subjects of the control group were been also selected among the personnel of the same hospital and so the control group was expected to present similar characteristics of cases. Therefore, the two groups of patients and controls would have similar distributions of all matching variables and hence, the possible confounding effect is expected to be minimal.

**Conclusion**

The results of this study indicated increased serum hs-CRP concentration in asthma compared to controls. Increased serum hs-CRP correlates positively with severity of asthma and thus can be considered as a tool in predicting asthma status. Regarding the association between serum CRP and future cardiovascular events, these findings highlight serum hs-CRP measurement in asthma not only in predicting asthma control but also for future development of cardiovascular morbidities. 

However, this topic requires further investigations particularly a longitudinal study in which changes in serum hs-CRP should be determined periodically concurrent with changes in clinical features over the course of asthma treatment.

## Notes

**Funding:** This study is a thesis project of Amin Hosseini (No: 416)
